# Suicide and Culture: A Reflection on Suicidal Behaviour Through Cultural Context

**DOI:** 10.1192/j.eurpsy.2023.2370

**Published:** 2023-07-19

**Authors:** O. Nombora, B. F. Silva, Â. Venâncio

**Affiliations:** 1 Psychiatry Department, Centro Hospitalar Vila Nova de Gaia/Espinho, EPE, Vila Nova de Gaia, Portugal

## Abstract

**Introduction:**

The cultural meaning of suicide has been gaining attention in mainstream psychiatry literature, as an attempt to comprehend the dynamic relationship between culture and suicidality. Moreover, an understanding of the sociocultural and contextual factors in the aetiology of suicidal behaviour is important to develop culturally appropriate suicide prevention and intervention strategies.

**Objectives:**

Through the lenses of critical cultural suicidology, we aim to reflect on the relationship between suicide and sociocultural aspects, emphasizing the importance of context, cultural meanings, and the role of culture in suicide research and prevention strategies.

**Methods:**

We conducted a qualitative review on the topic using PubMed database. Search terms used: *“suicidal behaviour”; “suicidal ideation”; “suicide”; “culture”; “cultural”.*

**Results:**

Studies revealed that culture might be significant to understand suicidal behaviour. Therefore, suicidologists have often referred to a cultural meaning of suicide. Several studies argue that qualitative studies that focus on the meanings of suicidal behaviour in different cultural contexts are more relevant for suicide prevention than much of the quantitative risk factor research that is currently being conducted. Scholars conceptualize culture as either a protective factor or a risk factor that shapes an individual’s likelihood of engaging in a suicidal act. To locate culture’s influence on suicidal behaviour is essential to begin with an examination of social interaction. The meanings of suicide from a group of people living in a cultural community might vary along subcultural groups and time. Thus, the meaning of suicide is dynamic rather than static. Such a view acknowledges culture as both occurring outside the person, as well as within the person and between persons. It provides a view of a dynamic relationship between the individual and his or her contextual circumstances in which the individual is not just a passive recipient of cultural influences but also an active meaning-making agent who interacts meaningfully with the environment.

**Conclusions:**

Although the medical view of suicidality is a dominant perspective in suicidology, understanding cultural dynamics in suicidality and the conceptualization of suicide as a culturally guided act, is crucial to better understand suicidal behaviours. Further studies are needed in order to understand this complex and dynamic relationship.

**Disclosure of Interest:**

None Declared

